# Whole-stream wastewater addition stimulates the green food web pathway but does not affect food chain length

**DOI:** 10.1007/s10750-025-06103-w

**Published:** 2026-01-22

**Authors:** Ioar de Guzman, Mario Brauns, Arturo Elosegi, Daniel von Schiller, Jose M. González, José M. Montoya, Aitor Larrañaga

**Affiliations:** 1 Department of Plant Biology and Ecology, Faculty of Science and Technology, University of the Basque Country (EHU), Barrio Sarriena s/n, 48940 Leioa, Spain; 2 Ecologie Comportementale Et Biologie Des Populations de Poissons (UMR Ecobiop), French National Research Institute for Agriculture, Food and Environment (INRAE), Saint-Pée Sur Nivelle, France; 3 Department of River Ecology, Helmholtz Centre for Environmental Research-UFZ, Magdeburg, Germany; 4 Departament de Biologia Evolutiva, Ecologia i Ciències Ambientals (BEECA), Universitat de Barcelona (UB), Av. Diagonal 643, 08028 Barcelona, Spain; 5 Institut de Recerca de l’Aigua (IdRA), Universitat de Barcelona (UB), Montalegre 6, 08001 Barcelona, Spain; 6 Instituto de Investigación en Cambio Global (IICG-URJC), Universidad Rey Juan Carlos, Tulipán s/n, 28933 Móstoles, Spain; 7 Departamento de Biología y Geología, Física y Química Inorgánica, Universidad Rey Juan Carlos (URJC), Tulipán s/n, 28933 Móstoles, Spain; 8 Centre for Biodiversity Theory and Modelling, Theoretical and Experimental Ecology Station, French National Center for Scientific Research, Moulis, France

**Keywords:** BACI experiment, Wastewater treatment plant, Food web, Stable isotopes, Green food web pathway, Food chain length

## Abstract

Most rivers in developed regions receive tertiary-treated effluents from wastewater treatment plants that still contain a complex mixture of pollutants, but their combined effects on food webs are poorly understood. We had the unique opportunity to divert treated effluent into a previously pristine stream and conduct a before-after/control-impact/paired experiment to quantify effluent effects on green and brown food web pathways (i.e., algae and detritus-based pathways) and total food chain length. We hypothesized that the increased biofilm biomass after the effluent addition would promote its consumption by primary consumers, thus favoring the green pathway. Moreover, we hypothesized that the increase in the primary production would lead to an increase in food chain length. Coarse detritus remained the main food resource for primary consumers, but the addition of the effluent promoted biofilm biomass and increased the relative importance of the green food web pathway. However, the effluent discharge did not impact food chain length. The present study reveals that properly treated and substantially diluted effluents can still affect food web pathways through changes in dietary composition of consumers. Thus, further reducing nutrient inputs from WWTPs would minimize the impacts of effluents on food webs.

**Supplementary Information:**

The online version contains supplementary material available at 10.1007/s10750-025-06103-w.

## Introduction

Environmental regulations such as the European Water Framework Directive or the North American Clean Water Act led to a worldwide implementation of waste water treatment plants (WWTP) to reduce sewage pollution entering freshwater ecosystems (Brion et al., 2015). Nevertheless, treated sewage still consists of a cocktail of pollutants, nutrients, organic matter, and pathogens (Pascual-Benito et al., 2020; Weitere et al., 2021). Some of these compounds are invariably toxic (Patel et al., 2020), whereas others, such as some nutrients, can promote biological activity but also become toxic at high concentrations (Wang et al., 2019). Therefore, the effects of treated effluents on freshwater ecosystems depend on their composition, which is determined by the inflowing sewage, as well as by the design and performance of the WWTPs (Roccaro, 2018), and on the dilution rate in the receiving water bodies (Büttner et al., 2022).

The strong effects of poorly treated and highly concentrated WWTP effluents on ecosystems are noticeable, such as the deterioration of water quality or the impairment of biological communities (Hamdhani et al., 2020). Given the complex nature of WWTP effluents, they may affect several components of freshwater communities simultaneously by altering both non-trophic and trophic interactions. However, the effects of wastewater on entire food webs have not received much attention (Brauns et al., 2022), and when addressed, contrasting responses have been reported. For instance, differing shifts in dietary contributions of resources and repercussions on food webs have been observed (Singer & Battin, 2007; Gücker et al., 2011; Baumgartner & Robinson, 2017). Some authors reported shifts from detritivory to herbivory downstream from WWTPs (Baumgartner & Robinson, 2017), thus switching the main energy source of the food webs and favoring the energy transfer through the green pathway (i.e., algae based) over the brown one (i.e., detritus based). These changes at the base of the food web can have important consequences on food webs as primary producers are more nutritious than detritus (Brett et al., 2017). Other studies reported a contribution of wastewater-derived organic matter to invertebrate diets below WWTPs (Singer & Battin, 2007; Gücker et al., 2011), but a consequent increase in secondary production was only reported by Singer and Battin (2007), who also observed shortened food chains in impacted reaches. The limited and contrasting knowledge of the effects of WWTP on food webs is exacerbated by the inherent limitations in the study design of most studies. The vast majority of studies addressing the effects of WWTP effluents on freshwater ecosystems adopt a Control:Impact design just comparing reaches upstream and downstream from the effluent inputs. Many of these studies have even been conducted years after the WWTPs were set into operation, and where communities might have adapted to the persistent stressors, reducing the effect size compared to situations shortly after the start of the WWTP operation. Moreover, as most WWTPs are located in urban areas, the upstream control reaches are often impacted by other stressors, which could mask the effects of WWTP effluents on ecosystem structure and functioning. Thus, this situation calls for sound experimental designs in unpolluted environments to isolate the effects of WWTP effluents.

Our efforts here complement prior works, where we had the unique opportunity to assess the effects of a treated WWTP effluent on a previously unpolluted stream by conducting a whole-ecosystem manipulation experiment with a BACIP (before-after/control-impact/paired) design (Downes et al., 2002). The effluent altered ecosystem functioning, leading to nutrient-enrichment-related responses as it subsidized primary producers and their exo-enzymatic activities but reduced their nutrient uptake capacity (Pereda et al., 2020). A laboratory experiment with effluent from the same WWTP suggested low toxicity for microbes and invertebrates, despite the reported concentrations of contaminants, as a general subsidy response was observed with increasing effluent concentration on microbial respiration, invertebrate RNA:body mass and growth rates rather than the predicted subsidy-stress response (Solagaistua et al., 2018). Consequently, in the field, the effluent altered community structure favoring the abundances of taxa tolerant to ecological degradation, but reducing the sensitive ones, and leading to more spatially heterogeneous communities (de Guzman et al., 2023; González et al., 2023). Moreover, using trophic link information collected in the literature, we estimated that the addition of the effluent altered the energy fluxes through food webs but it did not affect the efficiency with which energy was transferred (de Guzman et al., 2023). Although the latter approach proved to be able to point to the main changes in the trophic structure of the food web, it had a weakness: the trophic links distilled from the literature may not have been materialized in our system given the idiosyncratic properties of communities, such as the body size ranges of predators and preys, or the compartmentalization of the food web into the different microhabitats. To solve this uncertainty, in this study, we aimed to assess the effects of a treated and highly diluted WWTP effluent on different compartments of stream food webs by means of stable isotope analyses, which capture the realized trophic links in food webs. Nutrient-rich WWTP effluents can stimulate the productivity of freshwaters (Keck & Lepori, 2012; Pereda et al., 2019). Thus, we predicted that the WWTP effluent would increase the relevance of the green food web pathway (i.e., the food web chain that relies on algae) by increasing the contribution of primary producers into the diet of consumers induced by the enhanced biofilm biomass (Fig. S1). Concurrently, due to the increased energy availability at the base of the food web, the shifts in dietary contributions of consumers and the low toxicity of the effluent which did not lead to the disappearance of pollution-sensitive taxa, we predicted an increase in food chain length (Fig. S1), as more productive ecosystems can sustain longer food chains (Pimm, 1982).

## Materials and methods

### Study site and experimental design

The experiment was carried out in the Apraitz Stream (N Iberian Peninsula, 43°13′41.1″N 2°23′56.3″W, Fig. S1), a small, unpolluted stream with a mean discharge of 0.12 m^3^/s draining a 7 km^2^ catchment over sandstone and shale. The streambed in the experimental reach was dominated by cobbles and bedrock and surrounded by a young riparian forest, mainly composed of deciduous species such as black alder (*Alnus glutinosa* (L.) Gaertn.), hazel (*Corylus avellana* L.), and ash (*Fraxinus excelsior* L). The reach runs near the Apraitz WWTP, which releases the treated sewage into the Deba River through regular pulses (20–40 min every 2 h), 10 m upstream from the confluence between the Apraitz Stream and the Deba River. The WWTP receives urban and industrial sewage of approximately 90,000 population equivalents and treats, on average, 29,904 m^3^ of wastewater per day in its sequential biological reactors (https://www.acciona-agua.com). Sewage is mixed with activated sludge and subjected to aerobic and anaerobic conditions to reduce the load of organic matter and nitrogen before it receives tertiary treatment (precipitation with ferric sulfate) to reduce phosphorus concentration.

The experiment followed a BACIP design to control temporal and spatial variability and isolate the effect of the impact of interest with samples paired in time (Downes et al., 2002). Over the course of a year (May 2017 to May 2018), we diverted part of the WWTP effluent through a 125-mm-diameter pipe into the lowermost 150 m of the Apraitz Stream, with a final dilution rate similar to that of the effluent discharged into the Deba River (0.2–4% and 0.1–9% of effluent concentration, respectively; Pereda et al., 2020). We defined two 100-m long reaches: a control reach, upstream from the discharge and an impacted reach just below it. We sampled at both reaches in autumn, winter, and spring the year before and after the start of the effluent addition (May 2017).

### Effluent characteristics

Effluent characteristics (monitored by the managers of the WWTP), such as pH, conductivity, biochemical oxygen demand (BOD), chemical oxygen demand (COD), and total nitrogen (TN), differed among seasons, whereas total phosphorus did not vary (Table S1). Conductivity (678.8 ± 19.9 µS cm^−1^), COD (39.61 ± 2.40 mg L^−1^ O_2_), and TN (6.26 ± 0.57 mg L^−1^ NO_3_^−^) showed the highest values during autumn, and pH (7.12 ± 0.05) in winter and BOD (10.20 ± 0.97 mg L^−1^ O_2_) in spring (Table S1). During the study, *δ*^15^N_NO3_ was higher in the effluent (20.41‰ ± 4.38) than in ambient stream water (6.44‰ ± 0.37) (*n* = 5 and *n* = 2, respectively, 250 mL each, measurements following Bujak et al., 2021).

The effluent contributed, on average, 3.64% to stream discharge before the autumn sampling and 1.22 and 1.91% before the winter and spring samplings, respectively (Table S1, Pereda et al., 2020). The effluent discharge changed water physical and chemical characteristics significantly at the impact reach (Pereda et al., 2020). Dissolved oxygen saturation and pH decreased from around 100 to 92% and from 7.9 to 7.1, respectively. Electrical conductivity increased from around 280 to 427 μS cm^−1^, but water temperature remained unaffected. The concentrations of ammonium and soluble reactive phosphorus increased from 0.01 to 0.2 mg N/L and from 0.01 to 0.2 mg P L^−1^, respectively.

### Sampling and sample processing

#### Standing stock of basal food resources

Before starting the experiment, we deposited 50 artificial substrata (granite paving stones of 20 × 10 × 8 cm) along each reach (covering less than 0.5% of the streambed) to allow for biofilm colonization. In each sampling campaign and reach, we randomly chose five paving stones, scraped biofilms from their entire surface, and collected the slurry in filtered stream water (0.7-μm pore size, Whatman GF/F). In the laboratory, we homogenized the slurry and filtered subsamples of 20 ± 5 mL through pre-weighed filters (0.7-μm pore size), which were then oven-dried (70 °C, 72 h), weighed, combusted (500 °C, 5 h), and reweighed to obtain the ash-free dry mass (AFDM) per surface unit (g m^−2^) (Pereda et al., 2020). In addition, in each reach, we collected nine coarse detritus samples with a Surber sampler (0.09 m^2^, 0.5 mm mesh size), and we processed the organic matter of terrestrial origin retained on an 8-mm sieve. We separated leaves from the rest of the coarse detritus and processed each category to obtain AFDM in each sample (Pereda et al., 2020). Consumption of wood and other recalcitrant materials by macroinvertebrates is negligible compared to leaves (Díez et al., 2002), thus, we only considered leaves as a potential resource (hereafter coarse detritus).

#### Stable isotope analysis

We sampled consumers and their potential resources in each reach (control and impact) and occasion (autumn, winter, and spring, before and after effluent addition) for stable isotope analysis (SIA). We collected all available basal food resources, i.e., biofilm, fine detritus, coarse detritus (leaves from terrestrial vegetation), bryophytes, and filamentous green algae. Six composite biofilm samples were collected in each reach by scraping the whole surface of randomly picked cobbles and collecting the slurry in filtered river water (0.7 µm pore size, Whatman GF/F). Six samples of fine detritus were randomly collected per reach in each sampling campaign using a sediment corer (surface 81.7 cm^2^). The remaining basal resources were individually gathered from the riverbed. Macroinvertebrates were collected with a kick net (0.5 mm mesh size) in six longitudinal transects along each 100-m long reach, sorted, rinsed, and identified in the field to genus level (except for some Diptera identified to subfamily level and Annelida to subclass level) following Tachet et al. (2010). The identified invertebrates were assembled in up to nine samples per taxon and reach, containing from one to 55 individuals depending on their body mass. When possible, the digestive tract of the predators was removed, as gut contents can affect the isotopic signature (Mateo et al., 2008) and mollusks were extracted from their shells. Fish were sampled along the 100-m long reaches, enclosed with stop-nets, by depletion with a backpack-electrofishing unit with variable output current (MARTIN PESCADOR III, Alborlan S.L.). Up to five individuals per species and reach were caught, anesthetized with MS-222 and euthanized (reference number of the ethics commission of the University of the Basque Country: M20/2016/177). Samples of dorsal muscle were extracted, and samples were immediately frozen (− 20 °C) for SIA.

Samples of resources and consumers were freeze-dried (VirTis Benchtop 2 K) (from 12 to 72 h depending on water content), ground (Vibration MM301 ball-mill, Fisher Bioblock Scientific for resources, Precellys® 24 homogenizer, Bertin instruments for consumers), and weighed (approximately 1 mg for consumers, 10 mg for fine detritus, and 2 mg for other basal resources) into tin capsules (Lüdiwiss Sn 98, 5 × 8 mm) for SIA. Elemental concentrations of *δ*^13^C and *δ*^15^N were analyzed on a Flash 2000 elemental analyzer connected to a Delta V isotope ratio mass spectrometer operated in the continuous helium flow mode via ConFlo IV split interface (EA-IRMS; Thermo Fisher Scientific, Bremen, Germany). Three in-house standards were analyzed for quality assurance for every 15–16 samples. Results are reported as relative differences between ratios of samples and international standards (Pee Dee Belemnite for *δ*^13^C, atmospheric N for *δ*^15^N) and expressed in per mil delta notation [e.g., *δ*^13^*C* (‰) = (*Rsample*⁄*Rstandard* − 1)1000] (Fry, 2006). Analytical error (mean SD from in-house standards) associated with our sample runs was estimated at 0.2‰ for *δ*^13^C and 0.3‰ for *δ*^15^N.

### Data analysis

#### Contribution of resources to the diet of primary consumers

We estimated the contribution of basal resources to the diets of macroinvertebrate primary consumers at each sampling occasion and reach using *δ*^13^C and *δ*^15^N isotopic signatures as tracers with Bayesian Mixing Models (MixSIAR R package; Stock & Semmens, 2013). Autochthonous resources, coarse detritus, and fine detritus were treated as putative resources. We gathered all the collected leaf species within the coarse detritus category. We also collected biofilm, filamentous green algae and bryophytes within the sampling reaches despite the low abundance and sparse distribution of the latter ones, and combined them into the autochthonous resource category. The mixing models generate a distribution of possible mixing solutions based on the available resources, considering uncertainty and variation in consumers and trophic discrimination factor (TDF). Additionally, MixSIAR incorporates variation regarding sample processing and consumer variability (i.e., individual differences in digestibility, assimilation efficiency, and metabolic rates), providing error terms (Stock & Semmens, 2016). The TDF and uncertainties specific to aquatic invertebrates were used (0.1 ± 2.2‰ for *δ*^13^C and 2.6 ± 2.0‰ for *δ*^15^N; Brauns et al. (2018)). Concentration dependence (Phillips & Koch, 2002) and multiplicative error structure (Stock & Semmens, 2016) were also considered in the models. Posterior estimates of the proportional contribution of each resource to the diet of primary consumers were obtained for each reach through the Bayesian Mixing Models. Outliers within consumer signatures were previously checked through simulated mixing polygons (Smith et al., 2013) with the R packages “sp” (Pebesma & Bivand, 2005) and splancs (Bivand et al., 2017), and 10 out of 480 primary consumer samples were excluded. The method uses a Monte Carlo simulation to iterate Convex hulls (‘mixing polygons’) based on means and SD of source data and TDF. It applies the point-in-polygon assumption to test if source contributions can explain the consumer isotopic signature in the proposed mixing model.

#### Trophic position and maximum food chain length

Maximum food chain length, i.e., the linear trophic distance between basal resources and consumers at the top of the food web of each reach was estimated based on the maximum trophic position convention. Trophic positions (TP) of fish were assessed by comparing their *δ*^15^N values to the mean *δ*^15^N value of the basal resources at each sampling site (Cabana & Rasmussen, 1996): $TP_{ } = \frac{{\left( {\delta^{15} N_{{\text{top predator}}} - \delta^{15} N_{{{\mathrm{baseline}}}} } \right)}}{3.4} +$*ʎ*, where *3.4* is the TDF of *δ*^15^N (Vander Zanden & Rasmussen, 2001) and *ʎ* is the trophic level of the baseline indicator, set as 1 because primary producers were used as the baseline. Thus, the fish species with the highest TP on each occasion were used for the assessment of maximum food chain length (Table S2). We also tested if the WWTP effluents altered the TP of Adour minnow (*Phoxinus bigerri* Kottelat, 2007) and brown trout (*Salmo trutta* Linnaeus, 1758), the two fish species found in every sampling occasion and reach.

### Statistical analyses

All statistical analyses were performed using R software, version 3.6.0. (R Core Team, 2019). We conducted Linear Models with Period (before and after), Reach (control and impact), Season (autumn, winter and spring), and their interaction as factors to assess the effects of the effluent (Period:Reach, Period:Reach:Season) on the stock of basal resources, maximum food chain length, and trophic position of minnow and trout*.* The same factors were used for *δ*^13^C and *δ*^15^N of the entire community (from basal resources to fish) and *δ*^15^N of each functional group (basal resources, primary consumers, omnivores, predatory invertebrates, and fish) in Linear-Mixed Effects Models (function lme, in R package nlme (Pinheiro et al., 2020)) including also Taxon as a random factor. Consumer trophic groups were considered based on feeding preferences (de Guzman et al., 2023). Variance components of mixed effects models were estimated through restricted maximum likelihood and *p* values estimated using likelihood ratio tests (Pinheiro & Bates, 2006).

To test for the effect of the effluent on diet contribution analyses, we used Generalized Linear Models (GLMs) on the posterior estimates of the Bayesian models since including these variables in the Bayesian Mixing Models caused a lack of convergence. We included 9000 posterior estimates on diet contribution analyses for each resource and community (sampling occasion and reach). Posterior estimates related to diet contribution analyses were adjusted to a binomial distribution (link: logit) (Zuur et al., 2009). Eighteen GLMs were built for each variable using Period, Reach, Season, and their interactions as sources of variation, including all the possible combinations from the null model to the maximal model with the triple interaction term (Table S3). As the sample size was large, Bayesian Information Criterion (BIC) was used to penalize the size and select the best explanatory model in each case (Brewer et al., 2016). Model selection was made with the ‘modelsel’ function of the MuMIn package (Barton, 2020). Given the large number of posterior estimates generated through Bayesian modeling, violin plots were used to provide a more detailed visualization of the full distribution of the results.

Finally, we calculated the effect size of the effluent addition on the parameters of interest using mean values at each Period × Reach combination and using mean values at each Period-Reach in each Season to better show the seasonal variation as follows:

$$\text{Effect size }= \frac{\text{After }(\mathrm{Impact}/\mathrm{Control})}{\text{Before }(\mathrm{Impact}/\mathrm{Control})}$$

## Results

### Stock of basal resources

**Fig. 1:**
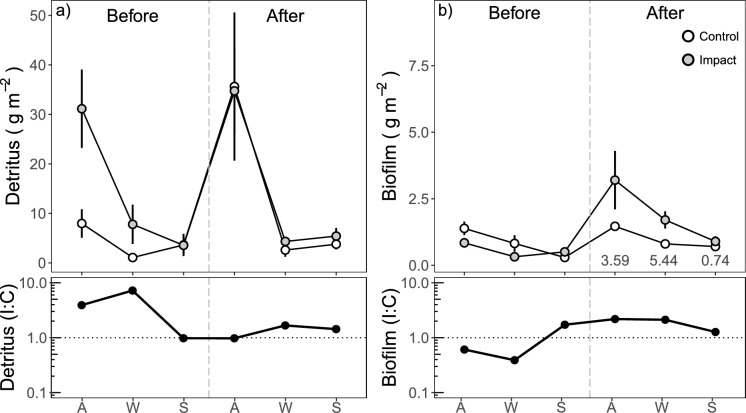
Mean (± SE) biomass of **a** detritus and **b** biofilm in the studied reaches before and after the addition of the effluent during autumn (A), winter (W), and spring (S). The vertical dashed line divides the before and after periods. Effect size for each season is shown when the interaction Period:Reach:Season was significant. Lower panels represent the impact to control ratios for detritus and biofilm. The horizontal dotted line represents same values for control and impact reaches

The standing stock of coarse detritus was up to two orders of magnitude larger than that of biofilm (Fig. 1). Coarse detritus stock was not affected by the effluent addition (Period:Reach: F_1,96_ = 2.07, *p* = 0.153 and Period:Reach:Season F_1,96_ = 0.76, *p* = 0.472), but differences in the stock were observed between periods (before vs. after), reaches (control vs. impact), and seasons (autumn vs. winter vs. spring) (Fig. 1a, Tables 1 and S3). The effluent addition significantly promoted biofilm biomass (Period:Reach: F_1,48_ = 7.17, *p* = 0.010, effect size 2.93; Fig. 1b, Tables 1 and S3) and affected the relative proportions of biofilm and detritus (effect size biofilm:CPOM ratio 5.06; Table S3). However, the effects of effluent on biofilm varied with season (Period:Reach:Season: F_2,48_ = 3.83, *p* = 0.029; effect sizes of 3.60 and 5.44 for autumn and winter, and 0.74 for spring).

**Table 1:** Linear model results of stock of basal resources, stable isotope ratios for the entire community, maximum food chain length, and trophic position of minnow and trout to assess the effects of effluent addition (Period:Reach, Period:Reach:Season)

	Coarse detritus				Biofilm			
	F	*p*	Coeff		F	*p*	Coeff	
Period	4.46	**0.037**	0.48 (A)		30.01	** < 0.001**	0.04 (A)	
Reach	12.22	**0.001**	0.54 (I)		0.88	0.353		
Season	34.05	** < 0.001**	− 0.55 (W)	− 0.39 (S)	18.19	** < 0.001**	− 0.32 (W)	− 0.66 (S)
Period:Reach	2.07	0.153			7.14	**0.010**	0.44 (A:I)	
Period:Season	0.67	0.512			0.53	0.589		
Reach:Season	1.30	0.279			0.83	0.442		
Period:Reach:Season	0.76	0.472			3.83	**0.029**	0.2 (A:I:W)	− 0.57 (A:I:S)
	*δ*^15^N*				*δ*^13^C*			
Period	95.17	** < 0.001**	1.18 (A)		3.34	0.068		
Reach	294.63	** < 0.001**	0.77 (I)		83.90	** < 0.001**	− 0.02 (I)	
Season	258.00	** < 0.001**	− 0.9 (W)	− 1.1 (S)	92.02	** < 0.001**	− 1.19 (W)	− 0.28 (S)
Period:Reach	54.15	** < 0.001**	1.3 (A:I)		0.39	0.534		
Period:Season	68.77	** < 0.001**	− 1.73 (A:W)	− 1.27 (A:S)	21.39	** < 0.001**	0.2 (A:W)	− 0.57 (A:S)
Reach:Season	2.44	0.087			9.94	** < 0.001**	0.76 (I:W)	0.54 (A:S)
Period:Reach:Season	1.94	0.144			2.69	0.068		
	Maximum food chain length				Trophic position_minnow_			
Period	5.08	**0.031**	0.49 (A)		11.19	**0.002**	0.41 (A)	
Reach	65.9	** < 0.001**	1.37 (I)		56.83	** < 0.001**	1.37 (I)	
Season	7.09	**0.003**	0.89 (W)	0.66 (S)	8.45	**0.001**	0.89 (W)	0.66 (S)
Period:Reach	1.03	0.317			0.54	0.466		
Period:Season	3.05	0.060			5.07	**0.010**	− 0.8 (A:W)	− 1.29 (A:S)
Reach:Season	3.12	0.057			1.31	0.280		
Period:Reach:Season	1.58	0.221			2.01	0.146		
	Trophic position _*trout*_							
Period	0.05	0.819						
Reach	3.55	0.068						
Season	16.96	** < 0.001**	0.96 (W)	0.34 (S)				
Period:Reach	0.02	0.876						
Period:Season	5.93	**0.006**	− 0.76 (A:W)	− 1.20 (A:S)				
Reach:Season	2.30	0.116						
Period:Reach:Season	1.62	0.212						

Linear mixed model results are marked with an asterisk and include the sample as random. Values in bold indicate statistical significance (*p* < 0.05). Coefficients are shown for significant responses and consider before Period, control Reach, and autumn Season as reference in all cases. A is after, I is impact, and W and S refer to winter and spring, respectively

### Stable isotope signatures

Effluent addition did not affect the entire community *δ*^13^C (Tables 1 and S4, Fig. S2) (Period:Reach: F_1,1921_ = 0.39, *p* = 0.534). However it increased community mean *δ*^15^N significantly (Period:Reach: F_1,1921_ = 54.15, *p* < 0.001, effect size 1.16; Tables 1 and S3, Fig. S2 and S3) with values rising from 5.89‰ ± 3.28 to 6.98‰ ± 0.18 in the impact site, whereas in the control site it only varied from 4.92‰ ± 2.97 to 5.06‰ ± 3.02 (Table S4). Similarly, the *δ*^15^N signature of individual consumer groups increased with the effluent (Table S5 and Fig. S3a–e, effect size 1.30 for primary consumers, 1.14 for omnivores, 1.15 for carnivores, and 1.09 for fish). Basal food resources were also enriched in ^15^N with the effluent (Table S6, effect size 1.23 for all basal resources, 1.40 for biofilm, 1.60 for autochthonous resources, and 1.34 for fine detritus), except for coarse detritus that did not vary (Table S6).

### Dietary contributions

**Fig. 2:**
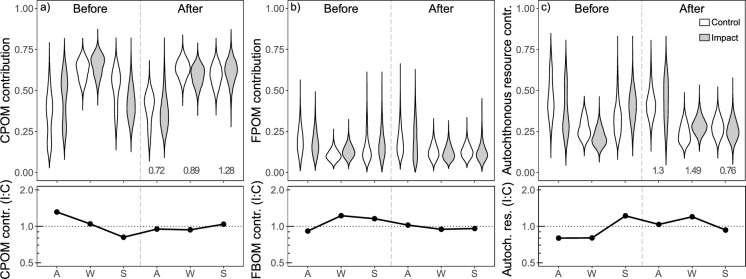
Bayesian posterior estimates showing the contribution of **a** coarse detritus (CPOM), **b** fine detritus (FPOM), and **c** autochthonous resources (biofilm, filamentous green algae, and bryophytes) to the diets of consumers in the studied reaches before and after the start of the effluent addition during autumn (A), winter (W), and spring (S). The width of each violin represents the relative density of the estimates at different values. The vertical dashed line divides the before and after periods. Effect size for each season is shown when full models were the preferred models. Lower panels represent the impact to control ratios for the contribution of each resource. The horizontal dotted line represents same values for control and impact reaches

Coarse detritus contributed the most (> 50%) to primary consumers’ diet, followed by autochthonous resources (means ranging between 22 and 46%) and fine detritus (< 20%) (Fig. 2, Table S7). With the effluent addition, the contribution of autochthonous resources on primary consumers’ diet increased (*p* < 0.001 and effect size of 1.12; Fig. 2c, Tables 2, S3 and S8), thereby reducing the contribution of coarse detritus (*p* < 0.001 and effect size of 0.95; Fig. 2a Tables 2, S3, and S8). However, autochthonous contributions varied with season and increased with the effluent in autumn and winter (effect sizes of 1.30 and 1.50, respectively), but decreased in spring (effect size of 0.76; Fig. 2c, Tables 2, S3, and S8). Coarse detritus followed the opposite trend, decreasing slightly in the presence of effluent (*p* < 0.001 and effect size of 0.95; Fig. 2a, Tables 2, S3, and S8). The contribution of coarse detritus decreased in autumn and winter (effect sizes of 0.82 and 0.79, respectively), but increased in spring (effect size of 1.28; Fig. 2a, Tables 2, S3, and S8). The dietary contribution of fine detritus was not affected by the effluent addition (Fig. 2b, Tables 2 and S8).

**Table 2:** Preferred models to explain the effect of the effluent addition on the contribution of basal resources (coarse detritus, fine detritus, and autochthonous resources) to primary consumers

Preferred model	Coarse detritus contr		Fine detritus contr	Autochthonous resource contr	
	Full model		Null model	Full model	
df	12		1	12	
logLik	− 59,242.56		− 17,906.6	− 48,142.48	
BIC	118,624.2		35,824.8	96,424	
	*p*	Coeff		*p*	Coeff
Period	< 0.001	0.18 (A)		< 0.001	− 0.16 (A)
Reach	0.803			0.112	
Season	< 0.001	1.15 (W), 0.7 (S)		< 0.001	− 0.84 (W), − 0.53 (S)
Period:Reach	< 0.001	− 0.54 (A:I)		< 0.001	0.44 (A:I)
Period:Season	< 0.001	− 0.15 (A:W), 0.12 (A:S)		< 0.001	0.01 (A:W), − 0.1 (A:S)
Reach:Season	< 0.001	− 0.33 (I:W), − 0.85 (I:S)		< 0.001	0.09 (I:W), 0.7 (I:S)
Period:Reach:Season	< 0.001	0.24 (A.I:W), 1.03 (A:I:S)		< 0.001	0.09 (A.I:W), − 0.86 (A:I:S)

*df* degrees of freedom, *logLik* log-likelihood ratios, *BIC* Bayesian Information Criterion are given. Coefficients of preferred models are shown when *p* < 0.05. Before Period and the control Reach and autumn Season are considered as a reference in all cases. A refers to after and I to impact, W to winter, and S to spring

### Maximum food chain length

**Fig. 3:**
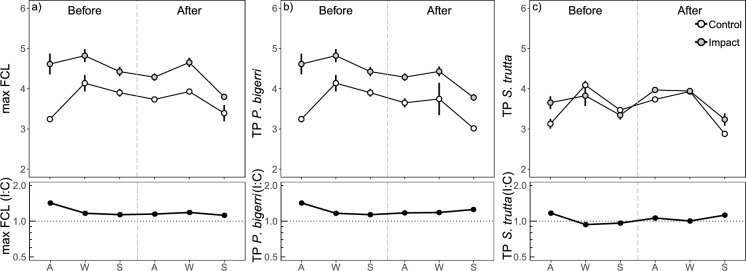
**a** Maximum food chain length (max FCL) and trophic position (TP) of **b** minnow (*P. bigerri*) and **c** trout (*S. trutta*) in the studied reaches before and after the start of the addition of the effluent during autumn (A), winter (W) and spring (S). The vertical dashed line divides the before and after periods. Lower panels represent the impact to control ratios for FCL and TP of the two species. The horizontal dotted line represents same values for control and impact reaches

From the five fish species sampled during the experiment, Adour minnow and brown trout were the species found in every occasion and reach (Table S2). Maximum food chain length and the trophic positions of these two species did not differ with the addition of the effluent (Fig. 3, Table 1). Maximum food chain length ranged between 3.24 ± 0.08 and 4.82 ± 0.16, and it was consistently larger in the impact reach (between 3.79 and 4.82) than in control (between 3.24 and 4.13) before and after effluent addition (Fig. 3a, Table 1). Minnow showed the highest trophic position in most of the sampling occasions compared to the other four species (Table S2). Overall, minnow showed a higher trophic position in the impact than in the control reach (Fig. 3b, Table 1). Trout showed similar values between reaches in each sampling occasion (Fig. 3c, Table 1, and S2).

## Discussion

Our whole-ecosystem manipulation experiment provided novel evidence for the effect of contemporary WWTP effluents on stream food webs. We demonstrated that even tertiary-treated and highly diluted effluent shifted the base of the food web and, consequently, modified the contribution of resources stimulating the green food web pathway. However, food chain length did not increase despite the higher availability of autochthonous resources.

### Effluent promoted biofilm and its dietary contribution

Overall, mixing models revealed coarse detritus of terrestrial origin as the main basal resource in our study site. Leaf litter is usually the major allochthonous input in forested headwater streams and, thus, constitutes the main energy and nutrient source for heterotrophic aquatic communities (Zhang et al., 2019) due to the limitation of primary production by canopy cover (Bernhardt et al., 2018) and dissolved nutrients (Elser et al., 2007). However, with the addition of the effluent, a trophic shift was observed whereby coarse detritus contribution decreased as the contribution of autochthonous resources was promoted. This drop in detritus contribution was evident in autumn (when the stream concentration of the effluent and the stock of the detritus were highest) and in winter, but the opposite was true for spring. There is evidence that increased dissolved nutrient availability for microorganisms promotes detritus quality and consumption (Gulis et al., 2006; Woodward et al., 2012). In this line, in a decomposition experiment conducted alongside this study, Pereda et al. (2020) found that the effluent addition increased invertebrate-driven leaf litter decomposition. However, de Guzman et al. (2023) showed no concomitant increase in the biomass of primary consumers in the same experimental setup. Therefore, the observation by Pereda et al. (2020) that invertebrate-driven litter decomposition increased without a parallel rise in the biomass of primary consumers or in the contribution of detritus to their diets suggests that enhanced resource consumption does not necessarily result into a greater assimilation by consumers.

In line with other studies (Pereda et al., 2019, 2021), in our experiment, biofilm biomass significantly increased with the effluent addition, showing an effect size of 2.92, and thus increasing its relative availability over the dominant coarse detritus with an effect size of 5.06 for the biofilm:coarse detritus ratio. The increase of biofilm biomass was paralleled by an increase in autochthonous resource contribution to the macroinvertebrate diet. Thus, these shifts suggest that effluent-derived nutrient inputs stimulated the green over the brown food web pathway by enhancing primary production and increasing the availability and quality of algal-based resources. Baumgartner & Robinson (2017) also assessed changes in dietary composition of primary consumers, who completely switched their mainly detritivorous diet (~ 100%) upstream from a WWTP towards herbivory in downstream reaches (> 80%). In a similar system, despite the larger availability of brown resources, Bumpers et al. (2017) also reported shifts from brown to green food web pathways with nutrient enrichment, as they observed a larger amount of herbivorous prey in the diet of predators.

### Community was enriched in ^15^N, but the maximum food chain length did not change

Although non-treated or primary-treated sewage are depleted in *δ*^15^N (di Lascio et al., 2013), secondary and tertiary-treated effluents are isotopically enriched in ^15^N due to the enzymatic preference of bacteria for the lighter ^14^N isotope during denitrification (Morrissey et al., 2013). In our study, the increased *δ*^15^N signature of the effluent was reflected in autochthonous resources, as well as in the entire community of the receiving stream. These results go in line with observations made in other studies, where autochthonous resources such as biofilm readily assimilate the WWTP-derived N (Baumgartner & Robinson, 2017). This enrichment at the base of the food web as a consequence of effluent addition was also transferred to the different groups of consumers, as has frequently been reported (Morrissey et al., 2013; Robinson et al., 2016; Baumgartner & Robinson, 2017).

Since in a previous work with the same setup, the low toxicity of the effluent did not lead to the extinction of pollution-sensitive taxa (González et al., 2023), we did not expect the effluent to shorten food chain length as reported in other studies (Singer & Battin, 2007). On the contrary, we expected that the increased energy available at the base of the food web, reflected by the higher contribution of autochthonous resources to the diet of consumers, would lengthen food chains as suggested in the productivity hypothesis (Pimm, 1982). However, we did not detect any variation in the maximum food chain length with the effluent. Although works in terrestrial ecosystems found evidence of the increase of food chain length with productivity (Arim et al., 2007; Young et al., 2013). Arim et al. (2007) reported that energy limitation could not necessarily be the determinant as many factors such as community composition, food web structure, or species abundance might respond to available energy. Moreover, some works in aquatic ecosystems have highlighted the importance of other factors, such as ecosystem size itself or resource availability (i.e., the productive space), being determinants of shaping the length of food chains (Post et al., 2000; Doi et al., 2009). In addition, in our study, the maximum food chain was unchanged as a result of effluent addition, being larger in the impact than in the control reach before and after the effluent addition. Turnover rates of *δ*^15^N in fish muscle are described to last a few months (between 30 and 85 days) (Miller, 2006; Ankjærø et al., 2012; Busst & Britton, 2018), time enough to detect changes in the duration of the current experiment.

Thus, the lack of effects on food chain length despite larger nutritious resource availability with the addition of effluent may be due to at least three reasons. First, minnows (i.e., the species with generally the highest trophic position in the study) from the impact reach may have moved to the adjacent and larger Deba River, which contains higher nutrient concentrations, foraging outside of the experimental reach and masking the potentially stimulating effects of higher resource standing stocks on food chain length. Nevertheless, this in-and-out movement from the reach was only possible for the individuals in the experimental reach. In contrast, the individuals in the control reach could only move down to the impact reach during high flow periods, but their return upstream to the control reach was highly unlikely given a barrier between the two reaches, a fact further supported by the presence of stone loaches exclusively in the impact reach. Along the same line, the lack of differences between trophic positions of trout between reaches could be driven by the greater mobility of this species together with the tendency of this species to eat terrestrial food resources (Erős et al., 2012), thus explaining both the lower trophic position and the lack of response to the effluent addition in this species. Second, although an increase in biofilm standing stock was reported in the experiment, de Guzman et al. (2023) did not observe an increase in primary consumer biomass with the effluent. This suggests that consumers were not resource-limited due to the large availability of detritus, partly explaining the lack of change in food chain length. Third, nutrient pollution can promote resources that are unsuitable for higher trophic levels, truncating energy flow and reducing food web efficiency (Davis et al., 2010). Thus, in the current experiment, the energy incorporated through the base of the green food web might have been trapped in a trophic dead end, for instance by the larger growth of less palatable algae, which may have buffered the effect on food chain length.

## Conclusions

The effects of the highly diluted WWTP effluents were small as alterations in the food chain length did not occur. However, significant shifts were observed in the food web towards the green pathway. Such alterations emerged only after employing a rigorous BACIP experimental design. Although the effects described seem to be weak, they are the response of a short-term exposure (1 year) and food web alterations may differ at longer temporal scales. The observed modifications on the food web architecture show that current advanced methods for treating polluted waters can still have detectable consequences on freshwater ecosystems, which might become relevant when managing high-quality ecosystems.

## Supplementary Information

Below is the link to the electronic supplementary material.

Supplementary file1 (DOCX 2368 KB)

## Data Availability

Data and code that support the findings of this study are openly available in Figshare and Github: 10.6084/m9.figshare.25428574.
