# Supplementary material for: Whole-stream wastewater addition stimulates the green food web pathway but does not affect food chain length
Source: Hydrobiologia. 2026 Jan 22;853(9):2681–94. doi: 10.1007/s10750-025-06103-w (PMC13035594; doi:10.1007/s10750-025-06103-w)
Supplement: Supplementary file 1 — Supplementary file1 (DOCX 2368 KB) [file 10750_2025_6103_MOESM1_ESM.docx]

## Journal: Hydrobiologia

## **Whole-stream wastewater addition stimulates the green food web pathway but does not affect food chain length**

Ioar de Guzman^1,2^, Mario Brauns^3^, Arturo Elosegi^1^, Daniel von Schiller^,4,5^, Jose M. González^6,7^, José M. Montoya^8^, Aitor Larrañaga^1^

^1^Department of Plant Biology and Ecology, Faculty of Science and Technology, University of the Basque Country (UPV/EHU), Barrio Sarriena s/n, 48940 Leioa, Spain

^2^ Ecologie Comportementale et Biologie des Populations de Poissons (UMR Ecobiop), French National Research Institute for Agriculture, Food and Environment (INRAE), Saint-Pée sur Nivelle, France

^3^Department of River Ecology, Helmholtz Centre for Environmental Research-UFZ, Magdeburg, Germany

^4^ Departament de Biologia Evolutiva, Ecologia i Ciències Ambientals (BEECA), Universitat de Barcelona (UB), Av. Diagonal 643, 08028 Barcelona, Spain

^5^ Institut de Recerca de l’Aigua (IdRA), Universitat de Barcelona (UB), Montalegre 6, 08001 Barcelona, Spain

^6^ Instituto de Investigación en Cambio Global (IICG-URJC), Universidad Rey Juan Carlos, Tulipán s/n, 28933 Móstoles, España

^7^Departamento de Biología y Geología, Física y Química inorgánica, Universidad Rey Juan Carlos (URJC), Tulipán s/n, 28933 Móstoles, Spain

^8^ Centre for Biodiversity Theory and Modelling, Theoretical and Experimental Ecology Station, French National Center for Scientific Research, Moulis, France

**Corresponding author :** Ioar de Guzman mirenioar.deguzman@ehu.eus

**Supplementary Tables**

**Table S1**. Physicochemical properties of the effluent and its contribution to the stream during the two previous months before each sampling occasion of the After period. BOD_5_ and COD are the Biochemical and Chemical Oxygen Demand, TP is Total Phosphorus, and TN is Total Nitrogen. Asterisks indicate the log-transformed parameters in linear models.

|  | Aug'17-Oct'17 | Nov'17-Jan'18 | Mar'18-May'18 | F | p |
| --- | --- | --- | --- | --- | --- |
| pH | 6.95 ± 0.05 (28) | 7.12 ± 0.05 (30) | 6.96 ± 0.06 (25) | 3.89 | **0.024** |
| Conductivity (µS/cm) | 678.80 ± 19.90 (28) | 446.80 ± 13.40 (31) | 610.90 ± 26.60 (25) | 38.30 | **<0.001** |
| BOD_5_* (mg/l O_2_) | 8.29 ± 0.60 (28) | 7.03 ± 0.63 (31) | 10.20 ± 0.97 (25) | 4.75 | **0.011** |
| COD* (mg/l O_2_) | 39.61 ± 2.40 (28) | 28.55 ± 1.66 (31) | 39.32 ± 4.29 (25) | 5.35 | **0.007** |
| TP* (mg/l PO_4_^3-^) | 1.38 ± 0.19 (10) | 1.34 ± 0.14 (9) | 1.61 ± 0.25 (10) | 0.27 | 0.765 |
| TN (mg/l NO_3_^-^) | 6.26 ± 0.57 (28) | 4.15 ± 0.63 (30) | 4.02 ± 1.32 (23) | 11.17 | **<0.001** |
| Effluent contribution (%) | 3.64± 0.03 (61) | 1.22 ± 0.07 (61) | 1.91 ± 0.09 (61) | 342.91 | **<0.001** |

**Table S2**. Trophic position (mean and standard error) of fish species in each reach. Values in bold belong to the species showing the maximum FCL at each reach. The number of individuals is shown in brackets. Periods Before (B) and After (A), Seasons Autumn (Au), Winter (W) and Spring (S), and Control (C) and Impact (I) Reaches are indicated.

|  |  |  | *Anguilla anguilla* | *Barbatula quignardi* | *Parachondrostoma arrigonis* | *Phoxinus bigerri* | *Salmo trutta* |
| --- | --- | --- | --- | --- | --- | --- | --- |
| B | Au | C | 3.11 ± 0.26 (4) |  |  | **3.24 ± 0.08 (5)** | 3.13 ± 0.12 (5) |
|  |  | I | 3.45 ± 0.14 (5) | 3.93 ± 0.12 (5) |  | **4.61 ± 0.26 (5)** | 3.65 ± 0.16 (5) |
|  | W | C | 3.94 ± 0.18 (3) |  |  | **4.14 ± 0.20 (5)** | 4.09 ± 0.1 (2) |
|  |  | I | 3.92 ± 0.23 (5) | 4.03 ± 0.04 (5) |  | **4.82 ± 0.16 (4)** | 3.83 ± 0.26 (5) |
|  | S | C | 3.74 ± 0.15 (5) |  |  | **3.90 ± 0.10 (5)** | 3.47 ± 0.01 (2) |
|  |  | I | 3.66 ± 0.03 (5) | 3.75 ± 0.09 (3) |  | **4.43 ± 0.11 (5)** | 3.34 ± 0.11 (8) |
| A | Au | C | 2.76 ± 0.18 (4) |  |  | 3.65 ± 0.11 (4) | **3.73 ± 0.06 (4)** |
|  |  | I | 3.14 ± 0.32 (4) | 3.74 ± 0.06 (5) |  | **4.29 ± 0.09 (5)** | 3.97 ± 0.06 (4) |
|  | W | C |  |  |  | 3.75 ± 0.40 (5) | **3.93 (1)** |
|  |  | I | 4.53 ± 0.07 (5) | 4.15 ± 0.10 (5) | **4.65 ± 0.12 (2)** | 4.43 ± 0.12 (5) | 3.94 (1) |
|  | S | C | **3.39 ± 0.20 (4)** |  |  | 3.02 ± 0.06 (5) | 2.88 ± 0.02 (4) |
|  |  | I | 3.79 ± 0.18 (5) | **3.798 (1)** |  | 3.78 ± 0.08 (5) | 3.24 ± 0.15 (5) |

**Table S3**. Effect size of treated effluent addition on the studied variables (Period:Reach interaction) considering all the samplings together or each season independently. Values in bold refer to statistically significant results. Variables with an asterisk have not been statistically tested.

| Variables | Effect size | | | |
| --- | --- | --- | --- | --- |
|  | Total | Autumn | Winter | Spring |
| Coarse detritus | 0.32 | 0.25 | 0.23 | 1.47 |
| Biofilm | **2.92** | **3.59** | **5.44** | **0.74** |
| Biofilm:Coarse detritus ratio* | 5.06 | 14.41 | 23.51 | 0.50 |
| CPOM contr. | **0.95** | **0.72** | **0.89** | **1.28** |
| FPOM contr. | 0.92 | 1.12 | 0.77 | 0.83 |
| Autochthonous resource contr. | **1.12** | **1.3** | **1.49** | **0.76** |
| δ^15^N | **1.16** | 1.14 | 1.22 | 1.15 |
| δ^13^C | 1.00 | 0.99 | 1.01 | 1.01 |
| δ^15^N _Basal resources_ | **1.24** | 1.44 | 1.11 | 1.28 |
| δ^15^N _Primary consumers_ | **1.31** | 1.35 | 1.44 | 1.11 |
| δ^15^N _Omnivores_ | **1.14** | **1.03** | **1.04** | **1.40** |
| δ^15^N _Carnivores_ | **1.15** | 1.13 | 1.11 | 1.20 |
| δ^15^N _Fish_ | **1.09** | 0.98 | 1.13 | 1.17 |
| δ^15^N _Autochthonous resources_ | **1.61** | 1.40 | 1.58 | 1.94 |
| δ^15^N _Biofilm_ | **1.40** | 1.34 | 1.20 | 2.06 |
| δ^15^N _Fine detritus_ | **1.35** | 1.09 | 1.49 | 2.06 |
| Max FCL | 0.94 | 0.81 | 1.02 | 0.99 |
| TP _minnow_ | 0.98 | 0.83 | 1.02 | 1.11 |
| TP _trout_ | 1.04 | 0.91 | 1.07 | 1.17 |

**Table S4**. Mean nitrogen (δ^15^N, ‰) and carbon stable isotope ratios (δ^13^C, ‰) of the entire community in the studied reaches in Autumn, Winter and Spring during the Before and After periods.

| Isotope | Season | δ^15^N | | δ^13^C | |
| --- | --- | --- | --- | --- | --- |
|  |  | Control | Impact | Control | Impact |
| Before | Autumn | 6.02 ± 3.15 | 6.99 ± 3.64 | -27.12 ± 1.91 | -27.10 ± 2.07 |
|  | Winter | 4.57 ± 3.00 | 5.60 ± 3.44 | -28.49 ± 1.69 | -27.55 ± 1.69 |
|  | Spring | 4.51 ± 2.59 | 5.45 ± 2.74 | -27.77 ± 1.62 | -27.03 ± 1.61 |
|  | Mean | 4.92 ± 2.97 | 5.89 ± 3.28 | -27.89 ± 1.81 | -27.22 ± 1.77 |
| After | Autumn | 7.19 ± 3.10 | 9.48 ± 4.24 | -27.24 ± 1.84 | -26.88 ± 1.91 |
|  | Winter | 3.82 ± 2.50 | 5.71 ± 3.44 | -28.39 ± 1.85 | -27.68 ± 1.87 |
|  | Spring | 4.40 ± 2.42 | 6.12 ± 3.13 | -28.04 ± 1.58 | -27.47 ± 1.69 |
|  | Mean | 5.06 ± 3.02 | 6.98 ± 3.95 | -27.91 ± 1.81 | -27.37 ± 1.85 |

**Table S5**. Linear mixed model results of δ^15^N for the groups considered with Period, Reach and Season as fixed factors and Sample as random. Values in bold indicate significant differences between factors (p < 0.05). Coefficients are shown for significant responses and consider Before Period, Control Reach and Autumn Season as reference in all cases. A is After, I is Impact, and W and S refer to Winter and Spring, respectively.

|  | Basal resources | | | | Primary consumers | | | | Omnivores | | | |
| --- | --- | --- | --- | --- | --- | --- | --- | --- | --- | --- | --- | --- |
|  | F | p | Coeff. | | F | p | Coeff. | | F | p | Coeff. | |
| Period | 0.76 | 0.385 |  |  | 93.16 | **<0.001** | 1.33 (A) |  | 69.96 | **<0.001** | 1.93 (A) |  |
| Reach | 7.92 | **0.005** | -0.61 (I) |  | 100.55 | **<0.001** | 0.46 (I) |  | 227.65 | **<0.001** | 1.48 (I) |  |
| Season | 9.97 | **<0.001** | -0.95 (W) | -1.58 (S) | 202.90 | **<0.001** | -1.93 (W) | -2.50 (S) | 139.55 | **<0.001** | -0.91 (W) | -0.37 (S) |
| Period:Reach | 4.65 | **0.032** | 1.22 (A:I) |  | 43.65 | **<0.001** | 2.04 (A:I) |  | 23.74 | **<0.001** | 1.54 (A:I) |  |
| Period:Season | 2.58 | 0.077 |  |  | 38.13 | **<0.001** | -2.17 (A:W) | -0.65 (A:S) | 61.91 | **<0.001** | -2.20 (A:W) | -2.37 (A:S) |
| Reach:Season | 2.16 | 0.117 |  |  | 3.02 | **0.0497** | -0.38 (I:W) | 0.12 (I:S) | 6.38 | **0.002** | 0.02 (I:W) | -1.00 (I:S) |
| Period:Reach:Season | 0.70 | 0.497 |  |  | 2.89 | 0.057 |  |  | 7.07 | **0.001** | -1.59 (A:I:W) | 0.13 (A:I:S) |
|  | Carnivores | | | | Fish | | | |  |  |  |  |
|  | F | p | Coeff. | | F | p | Coeff. | |  |  |  |  |
| Period | 32.32 | **<0.001** | 1.41 (A) |  | 0.12 | 0.730 |  |  |  |  |  |  |
| Reach | 66.59 | **<0.001** | 1.45 (I) |  | 68.24 | **<0.001** | 1.76 (I) |  |  |  |  |  |
| Season | 166.14 | **<0.001** | -1.07 (W) | -1.20 (S) | 8.82 | **<0.001** | 1.08 (W) | 0.23 (S) |  |  |  |  |
| Period:Reach | 16.49 | **<0.001** | 1.79 (A:I) |  | 4.41 | **0.037** | -0.14 (A:I) |  |  |  |  |  |
| Period:Season | 24.77 | **<0.001** | -1.77 (A:W) | -1.73 (A:S) | 6.75 | **0.002** | -2.37 (A:W) | -2.54 (A:S) |  |  |  |  |
| Reach:Season | 15.95 | **<0.001** | -1.48 (I:W) | -1.06 (I:S) | 0.42 | 0.658 |  |  |  |  |  |  |
| Period:Reach:Season | 1.08 | 0.340 |  |  | 1.57 | 0.212 |  |  |  |  |  |  |

**Table S6**. Linear models and linear mixed model results of δ^15^N for the basal resources with Period, Reach and Season as sources of variation (and Sample as random factor for mixed models indicated with an asterisk). Autochthonous resources include biofilm, filamentous green algae and bryophytes. Values in bold indicate significant differences between factors (p < 0.05). Coefficients are shown for significant responses and consider Before Period, Control Reach and Autumn Season as reference in all cases. A is After, I is Impact, and W and S refer to Winter and Spring, respectively.

|  | Biofilm | | | Autochthonous resources | | |
| --- | --- | --- | --- | --- | --- | --- |
|  | F | p | Coeff. | F | p | Coeff. |
| Period | 26.28 | **<0.001** | -0.01 (A) | 2.48 | 0.118 |  |
| Reach | 36.99 | **<0.001** | 0.12 (I) | 7.22 | **0.008** | -0.67 (I) |
| Season | 98.16 | **<0.001** | -1.11 (W), -3.30 (S) | 10.31 | **<0.001** | -1.24 (W), -2.98 (S) |
| Period:Reach | 21.55 | **<0.001** | 2.01 (A:I) | 11.37 | **0.001** | 1.78 (A:I) |
| Period:Season | 2.23 | 0.117 |  | 4.62 | **0.012** | 0.33 (A:W), 1.75 (A:S) |
| Reach:Season | 0.36 | 0.702 |  | 1.01 | 0.369 |  |
| Period:Reach:Season | 1.12 | 0.333 |  | 0.12 | 0.886 |  |
|  | Fine detritus | | | Coarse detritus | | |
|  | F | p | Coeff. | F | p | Coeff. |
| Period | 4.45 | **0.039** | 2.17 (A) | 2.69 | 0.103 |  |
| Reach | 24.17 | **<0.001** | 0.77 (I) | 1.08 | 0.301 |  |
| Season | 36.03 | **<0.001** | 0.59 (W), -0.04 (S) | 2.44 | 0.090 |  |
| Period:Reach | 6.93 | **0.011** | 1.41 (A:I) | 0.67 | 0.414 |  |
| Period:Season | 34.37 | **<0.001** | -3.34 (A:W), -3.00 (A:I) | 0.54 | 0.585 |  |
| Reach:Season | 3.34 | **0.043** | 0.32 (I:W), -0.97 (I:S) | 2.69 | 0.071 |  |
| Period:Reach:Season | 0.56 | 0.576 |  | 0.54 | 0.586 |  |

**Table S7**. Mean values and credible intervals (upper and lower intervals within brackets) of each resource's contribution to primary consumers' diet.

|  |  | Coarse detritus | | Fine detritus | | Autochthonous resources | |
| --- | --- | --- | --- | --- | --- | --- | --- |
|  |  | Control | Impact | Control | Impact | Control | Impact |
| Before | Autumn | 0.35 (0.08 - 0.58) | 0.46 (0.18 - 0.69) | 0.19 (0.04 - 0.35) | 0.18 (0.04 - 0.32) | 0.46 (0.23 - 0.71) | 0.37 (0.14 - 0.65) |
|  | Winter | 0.63 (0.50 - 0.74) | 0.66 (0.52 - 0.77) | 0.11 (0.04 - 0.17) | 0.13 (0.06 - 0.21) | 0.27 (0.17 - 0.39) | 0.22 (0.12 - 0.32) |
|  | Spring | 0.52 (0.27 - 0.71) | 0.42 (0.23 - 0.61) | 0.15 (0.02 - 0.33) | 0.17 (0.03 - 0.35) | 0.33 (0.16 - 0.55) | 0.41 (0.22 - 0.60) |
| After | Autumn | 0.39 (0.17 - 0.56) | 0.37 (0.17 - 0.59) | 0.19 (0.06 - 0.36) | 0.20 (0.02 - 0.41) | 0.42 (0.25 - 0.61) | 0.43 (0.16 - 0.68) |
|  | Winter | 0.63 (0.52 - 0.74) | 0.59 (0.47 - 0.70) | 0.13 (0.05 - 0.22) | 0.12 (0.05 - 0.21) | 0.24 (0.14 - 0.36) | 0.29 (0.19 - 0.40) |
|  | Spring | 0.59 (0.45 - 0.72) | 0.62 (0.47 - 0.76) | 0.13 (0.05 - 0.21) | 0.12 (0.03 - 0.23) | 0.28 (0.17 - 0.39) | 0.26 (0.14 - 0.38) |

**Table S8**. Model-selection to explain the effect of the effluent addition on basal resource (coarse detritus, fine detritus and autochthonous resources) contribution to primary consumers. Degrees of freedom (df), log-likelihood ratios (logLik), Bayesian Information Criterion (BIC), and the difference with the model with lowest value (∆BIC) are given. Models with the lowest BIC are shown in bold. Coefficients related to the effect of the effluent are shown for the best model. Period (P), Reach (R) and Season (S) are included as factors. The models tested were: null model, three models with a single source of variation, three models with two sources of variation, three models with two sources of variation and their interaction term, three models with three sources of variation and one double interaction term, two models with the three sources of variation and two double interaction terms, a model with the three sources of variations and the three double interaction terms and the maximal model, which also included the triple interaction term.

|  | Model | P | R | S | P:R | P:S | R:S | P:R:S | df | logLik | BIC | ∆BIC | P(A):R(I) | P(A):R(I):S(W) | P (A):R (I):S (Sp) |
| --- | --- | --- | --- | --- | --- | --- | --- | --- | --- | --- | --- | --- | --- | --- | --- |
| Coarse detritus contribution | **glm0** | **+** | **+** | **+** | **+** | **+** | **+** | **+** | **12** | **-59242.56** | **118624.2** | **0** | **-0.54** | **0.24** | **1.03** |
|  | glm2 | + | + | + |  | + | + |  | 9 | -60023.27 | 120150.8 | 1526.64 |  |  |  |
|  | glm1 | + | + | + | + | + | + |  | 10 | -60039.27 | 120194.4 | 1570.24 |  |  |  |
|  | glm10 | + |  | + |  | + |  |  | 6 | -60518.04 | 121105.6 | 2481.41 |  |  |  |
|  | glm7 | + | + | + |  | + |  |  | 7 | -60519.58 | 121120.3 | 2496.08 |  |  |  |
|  | glm4 | + | + | + | + | + |  |  | 8 | -60536.61 | 121165.9 | 2541.73 |  |  |  |
|  | glm5 | + | + | + |  |  | + |  | 7 | -62031.7 | 124144.5 | 5520.33 |  |  |  |
|  | glm3 | + | + | + | + |  | + |  | 8 | -62045.91 | 124184.5 | 5560.33 |  |  |  |
|  | glm8 |  | + | + |  |  | + |  | 6 | -62293.57 | 124656.7 | 6032.47 |  |  |  |
|  | glm13 | + |  | + |  |  |  |  | 4 | -62519.9 | 125086.2 | 6461.95 |  |  |  |
|  | glm6 | + | + | + | + |  |  |  | 6 | -62538.71 | 125147 | 6522.76 |  |  |  |
|  | glm16 |  |  | + |  |  |  |  | 3 | -62781.08 | 125596.9 | 6972.73 |  |  |  |
|  | glm11 |  | + | + |  |  |  |  | 4 | -62782.62 | 125611.6 | 6987.39 |  |  |  |
|  | glm14 | + |  |  |  |  |  |  | 2 | -73768.1 | 147559.4 | 28935.18 |  |  |  |
|  | glm12 | + | + |  |  |  |  |  | 3 | -73769.58 | 147573.9 | 28949.73 |  |  |  |
|  | glm9 | + | + |  | + |  |  |  | 4 | -73787.78 | 147621.9 | 28997.72 |  |  |  |
|  | glm17 |  |  |  |  |  |  |  | 1 | -74011.72 | 148035 | 29410.83 |  |  |  |
|  | glm15 |  | + |  |  |  |  |  | 2 | -74013.2 | 148049.6 | 29425.38 |  |  |  |
| Fine detritus contribution | **glm17** |  |  |  |  |  |  |  | **1** | **-17906.6** | **35824.8** | **0** |  |  |  |
|  | glm15 |  | + |  |  |  |  |  | 2 | -17906.29 | 35835.8 | 10.96 |  |  |  |
|  | glm14 | + |  |  |  |  |  |  | 2 | -17908.93 | 35841 | 16.25 |  |  |  |
|  | glm12 | + | + |  |  |  |  |  | 3 | -17908.62 | 35852 | 27.21 |  |  |  |
|  | glm9 | + | + |  | + |  |  |  | 4 | -17910.58 | 35867.5 | 42.72 |  |  |  |
|  | glm16 |  |  | + |  |  |  |  | 3 | -17947.69 | 35930.2 | 105.37 |  |  |  |
|  | glm11 |  | + | + |  |  |  |  | 4 | -17947.39 | 35941.1 | 116.35 |  |  |  |
|  | glm13 | + |  | + |  |  |  |  | 4 | -17950.07 | 35946.5 | 121.71 |  |  |  |
|  | glm8 |  | + | + |  |  | + |  | 6 | -17949.66 | 35968.9 | 144.08 |  |  |  |
|  | glm10 | + |  | + |  | + |  |  | 6 | -17951.25 | 35972 | 147.26 |  |  |  |
|  | glm6 | + | + | + | + |  |  |  | 6 | -17951.77 | 35973.1 | 148.29 |  |  |  |
|  | glm7 | + | + | + |  | + |  |  | 7 | -17950.95 | 35983 | 158.24 |  |  |  |
|  | glm5 | + | + | + |  |  | + |  | 7 | -17952.04 | 35985.2 | 160.43 |  |  |  |
|  | glm4 | + | + | + | + | + |  |  | 8 | -17952.96 | 35998.6 | 173.84 |  |  |  |
|  | glm3 | + | + | + | + |  | + |  | 8 | -17954.03 | 36000.8 | 175.98 |  |  |  |
|  | glm2 | + | + | + |  | + | + |  | 9 | -17953.23 | 36010.8 | 185.99 |  |  |  |
|  | glm1 | + | + | + | + | + | + |  | 10 | -17955.24 | 36026.4 | 201.58 |  |  |  |
|  | glm0 | + | + | + | + | + | + | + | 12 | -17955.06 | 36049.2 | 224.41 |  |  |  |
| Autochthonous resource contribution | **glm0** | **+** | **+** | **+** | **+** | **+** | **+** | **+** | **12** | **-48142.48** | **96424** | **0** | **0.44** | **0.09** | **-0.86** |
|  | glm4 | + | + | + | + | + |  |  | 8 | -48358.15 | 96809 | 384.98 |  |  |  |
|  | glm1 | + | + | + | + | + | + |  | 10 | -48353.25 | 96822.4 | 398.37 |  |  |  |
|  | glm10 | + |  | + |  | + |  |  | 6 | -48415.63 | 96900.8 | 476.75 |  |  |  |
|  | glm7 | + | + | + |  | + |  |  | 7 | -48431.68 | 96944.5 | 520.45 |  |  |  |
|  | glm2 | + | + | + |  | + | + |  | 9 | -48437.04 | 96978.4 | 554.34 |  |  |  |
|  | glm6 | + | + | + | + |  |  |  | 6 | -48619.82 | 97309.2 | 885.13 |  |  |  |
|  | glm3 | + | + | + | + |  | + |  | 8 | -48624.85 | 97342.4 | 918.39 |  |  |  |
|  | glm13 | + |  | + |  |  |  |  | 4 | -48677.04 | 97400.4 | 976.4 |  |  |  |
|  | glm5 | + | + | + |  |  | + |  | 7 | -48699.07 | 97479.3 | 1055.22 |  |  |  |
|  | glm16 |  |  | + |  |  |  |  | 3 | -48788.58 | 97611.9 | 1187.88 |  |  |  |
|  | glm11 |  | + | + |  |  |  |  | 4 | -48804.57 | 97655.5 | 1231.45 |  |  |  |
|  | glm8 |  | + | + |  |  | + |  | 6 | -48810.66 | 97690.9 | 1266.83 |  |  |  |
|  | glm9 | + | + |  | + |  |  |  | 4 | -51705.65 | 103457.7 | 7033.62 |  |  |  |
|  | glm14 | + |  |  |  |  |  |  | 2 | -51760.77 | 103544.7 | 7120.68 |  |  |  |
|  | glm12 | + | + |  |  |  |  |  | 3 | -51776.47 | 103587.7 | 7163.67 |  |  |  |
|  | glm17 |  |  |  |  |  |  |  | 1 | -51868.86 | 103749.3 | 7325.26 |  |  |  |
|  | glm15 |  | + |  |  |  |  |  | 2 | -51884.55 | 103792.3 | 7368.25 |  |  |  |

## **Supplementary Figures**


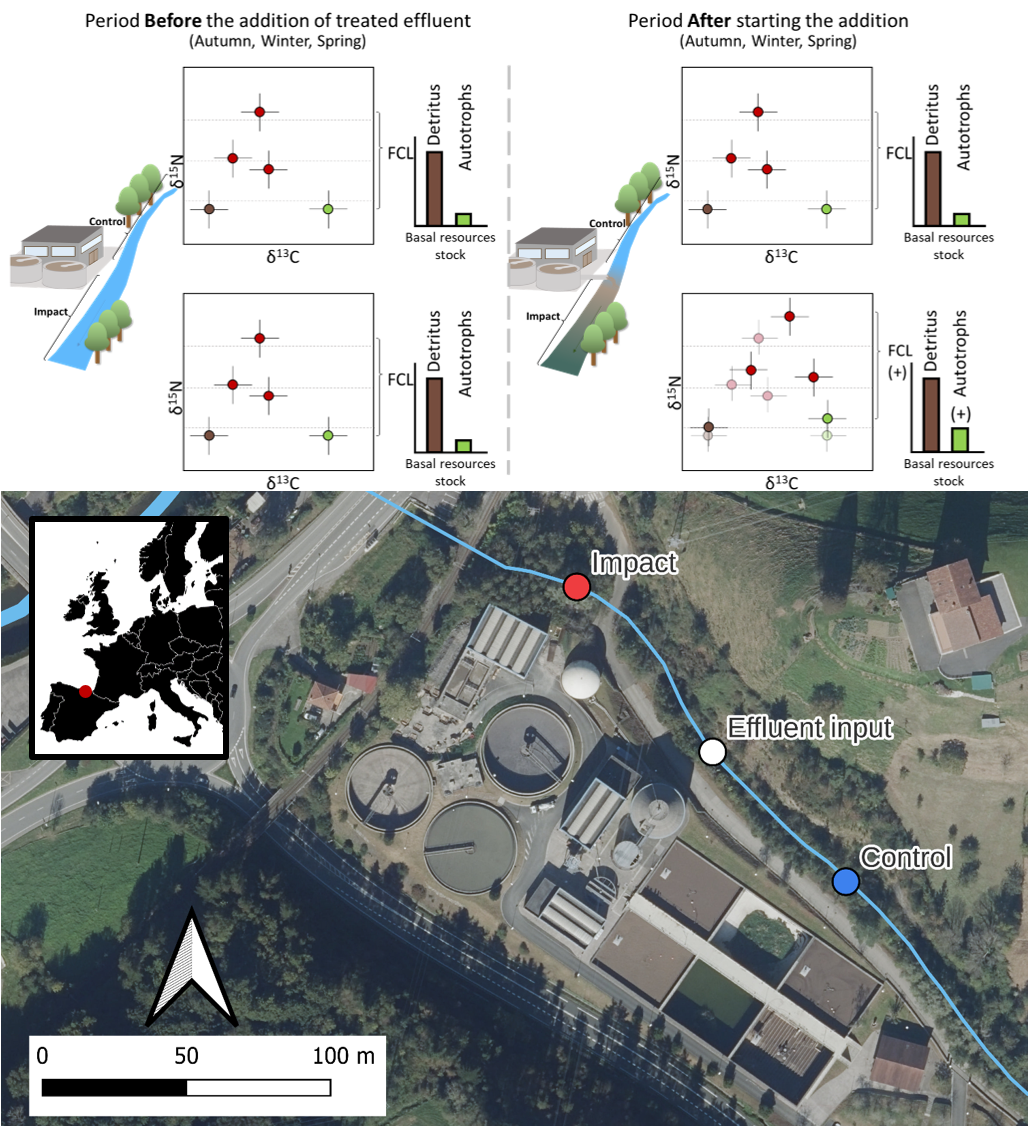


**Fig. S1** Schematic drawing of the BACIP experimental design with a conceptual figure of the proposed hypotheses, and a map of the study site. The Control reach is upstream from the effluent pouring location. The Impact site receives treated effluent during the After period. FCL refers to food chain length. The food web arrangement of the C-N biplot in the scenarios without effluent input is shown as reference in lighter colours in the Impact-After biplot. δ^15^N indicates the trophic position of each element of the biplot, whereas δ^13^C informs about the relative proximity to the basal resources and thus on their contribution to the diet of consumers. (+) symbol indicates an increase. Increased biofilm biomass and contribution of primary producers into the diet of consumers are expected with the addition of the effluent, as well as an increase in FCL.


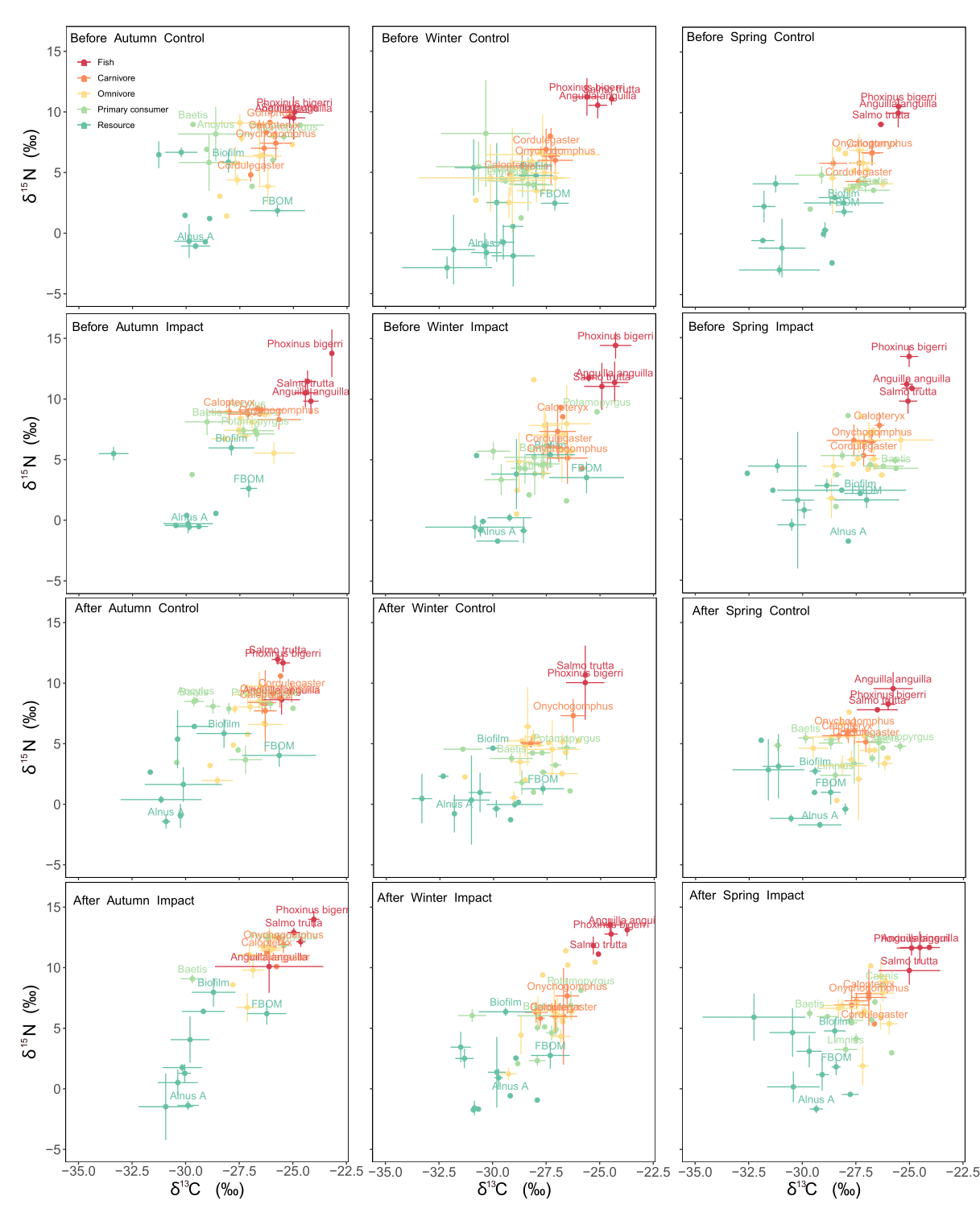


╔

**Fig. S2.** Stable isotope biplot of δ^15^N and δ^13^C showing mean (± SD) isotope signatures for each taxon.





**Fig. S3**. Nitrogen stable isotope ratios (δ^15^N, ‰) of (a) basal resources, (b) biofilm, (c) primary consumers, (d) omnivores, (e) predatory invertebrates and (f) fish in the studied reaches in Autumn (A), Winter (W) and Spring (S) during the Before and After periods. The box plots show the median, the interquartile range, and the tails of the distribution, and dots represent outliers. Coefficients for the Period:Reach interaction obtained from separate models for each season are shown when the interaction Period:Reach:Season was significant.
